# Antibiotic Stewardship in Community Pharmacies: A Scoping Review

**DOI:** 10.3390/pharmacy6030092

**Published:** 2018-08-23

**Authors:** Shazia Jamshed, Fadzlan Padzil, Siti Hadijah Shamsudin, Siti Halimah Bux, Abdul Aziz Jamaluddin, Akshaya Srikanth Bhagavathula, Saira Azhar, Mohamed Azmi Hassali

**Affiliations:** 1 Department of Pharmacy Practice, International Islamic University, Kuantan Pahang 25200, Malaysia; fad21an2190@gmail.com (F.P.); shadijah@iium.edu.my (S.H.S.); sitihalimah@iium.edu.my (S.H.B.); azizjamaludin2003@yahoo.com (A.A.J.); 2 Department of Internal Medicine, College of Medicine and Health Sciences, United Arab Emirates University, Al Ain, Abu Dhabi 15551, UAE; akshaypharmd@gmail.com; 3 College of Pharmacy, Princess Nourah Binti Abdul Rahman University, Riyadh 11671, Saudi Arabia; SAAzhar@pnu.edu.sa; 4 Discipline of Social and Administrative Pharmacy, School of Pharmaceutical Sciences, Universiti Sains Malaysia, Penang 11800, Malaysia; azmihassali@usm.my

**Keywords:** antibiotic dispensing, antibiotic stewardship, perceptions, attitudes, community pharmacists, community pharmacies

## Abstract

The increase in antibiotic resistance has frequently been linked to unrestrained antibiotic dispensing. This review was conducted to mainly assess the perception and attitudes of community pharmacists towards antibiotic dispensing. This scoping review was performed between June 2016 and September 2016 to identify published studies related to the perception and attitudes of community pharmacists towards antibiotic dispensing. The combination of terms such as ‘antibiotic dispensing’, ‘antimicrobial resistance’, ‘community pharmacy’, and ‘community pharmacists’ were searched in electronic databases such as PubMed, ProQuest, Google Scholar, and Science Direct. Only published articles within the last 12 years were included. A total of 13 studies were identified. In general, community pharmacists have good awareness and knowledge of antibiotic dispensing. However, the majority of them are still selling antibiotics to their customers and/or patients for unjustified reasons. The community pharmacists seem well aware of the antimicrobial resistance crisis and considered it a significant health issue. However, many embraced the concept that dispensing antibiotics without medical prescription (DAwMP) can be one of the key features in the dissemination of multidrug resistant bacteria.

## 1. Introduction

Increased prevalence of drug-resistant bacteria leads to an upsurge in morbidity and mortality from bacterial infections [1]. Infection thrives due to drug-resistant bacteria and claims thousands of lives each year [2]. A few commonly-encountered bacterial strains that develop resistance to antimicrobials are *Escherichia coli (E. coli)*, *Klebsiella pneumoniae*, *Streptococcus pneumonia*, and methicillin-resistant *Staphylococcus aureus* (MRSA) [3].

Globally, one of the key issues in tuberculosis (TB) treatment is drug resistance. The statistics on anti-TB drug resistance highlighted 123,000 patients with MDR-TB or rifampicin-resistant tuberculosis (RR-TB) and around three-fourths were located in India, South Africa, China, and European regions [4]. The report from the Centre for Disease Dynamics, Economics, and Policy (CDDEP) outlined an interesting scenario for MRSA.

During 2014 and 2015 it was reported that the incidence for MRSA was declined in United States (44%), Europe (18%) Canada (16%), and South Africa (28%) but it gained momentum in India (40%), Latin America (90%), Australia, and sub-Saharan Africa [5,6,7,8,9]. 

*E. coli* emerged as difficult-to-treat extended-spectrum betalactamase (ESBL) producers exhibiting resistance to newer third-generation cephalosporins. In 2013, 17 European countries reported the majority of *E. coli* isolates (85–100%) as ESBL-positive [6]. In the Asian region, 11 countries reported 28% of the *E. coli* family as ESBL-positive (UTIs) followed by resistance to both third- and fourth-generation cephalosporins [10]. Related to Enterobacteriaceae, five countries of Europe reported having increased incidence of Carbapenem-resistant Enterobacteriaceae (CRE) in 2013 [6], while in the United States, 11% of *K. pneumoniae* and 2% of *E. coli* were resistant to carbapenem [2].

The root cause of this problem is multifactorial and stems from the overuse of antibiotics, inappropriate antibiotic prescribing and dispensing, extensive use in agriculture and veterinary sectors, lack of new antibiotics, and weak regulatory barriers [11]. There is no denying that the problem of antimicrobial resistance is burdening both the developed and developing regions, being further aggravated by the nonprescription use of antibiotics [12,13,14]. Likewise, the problem of dispensing of antibiotics without prescription is also observed in many regions, except for US, Canada, and Northern Europe [13,15]. It is difficult to ascertain the comprehensive effect of antimicrobial resistance, but in the middle- and lower-income regions judicious sale of antibiotics is always a question mark [16,17,18,19,20,21,22,23,24,25,26,27,28].

An amalgamation of program elements focused on attitude and behavior changes is vital to achieve optimal health outcomes in a population. Antimicrobial stewardship (AMS) is an intervention program directed to improve and sustain appropriate antibiotic use in the absence of antimicrobial resistance and strengthen patient safety in a cost-effective manner [29]. The American Society of Health System Pharmacists outlined pharmacists as appropriate antimicrobial stewards who can responsibly acquire projected roles in antimicrobial stewardship programs and can exercise profound influence through participatory action in infection prevention and control measures [30]. Precisely, the successful execution and maintenance of this program depend on the knowledge and attitudes of the pharmacists working in both hospital and community settings. Globally, community pharmacists are well-documented to maintain reinforced care and services with customers and/or patients and, thus, are in well-placed positions to implement interventions related to stewardship and medication management in both minor and major conditions. 

This scoping review focused on the perception and attitude of community pharmacists towards antibiotic dispensing without prescription and the related factors and facilitators with most sought-after antibiotics in different diseases. The review is also expected to highlight research gaps followed by recommendations of interventions and health education.

## 2. Materials and Methods

A step-wise methodological framework by Arksey and O’Malley was the basis of this scoping review [31]. The step-wise framework helps in identifying the research question and search for the relevant studies followed by the selection of studies and projecting the data into chart form for the ease of collation, summary, and concluding the results. This framework serves as a methodological basis only, and the methodology was strengthened further by consulting the work of Levac, Colquhoun, and O’Brien [32] and studied their recommendations for each stage of framework. This ultimately helps in the precision of the scoping study methodology. 

The framework steps taken were (1) research questions identification; (2) relevant literature identification; (3) screening and selection of relevant literature; (4) data charting; and (5) analyzing summarizing, and reporting the results.

### 2.1. Step 1: Identification and Development of Research Question

As this review mainly focused on the perceptions and attitudes towards antibiotics dispensing among community pharmacists, the research question was “What are the opinions and attitudes of community pharmacists on antibiotic sales and does it affect the antibiotic resistance?”

We identified five areas of interest based on the antibiotic sales:

(a) What are the perceptions of community pharmacists towards antibiotic dispensing?
(b) What are the attitudes of community pharmacists towards antibiotic dispensing?
(c) How frequently did community pharmacists sell antibiotics in community pharmacies?
(d) How often did customers demand antibiotics in community pharmacies?
(e) What is the suitability of antibiotics that were dispensed from community pharmacies?

### 2.2. Step 2: Relevant Literature Identification

A literature search was done from June 2016 until September 2016 to find and classify published studies related to knowledge, perception, and attitudes of community pharmacists towards antimicrobial dispensing. The search was performed using Boolean operators for the following combination: “perceptions”, “attitudes”, “antibiotic”, “antibiotic resistance”, “antibiotic dispensing”, “community pharmacy”, and “community pharmacists”. The electronic databases used for searching include PubMed, ProQuest, Google Scholar, and Science Direct.

### 2.3. Step 3: Screening and Selection of Relevant Literature

The articles were reviewed by reading the titles and the abstracts. Studies that used qualitative or quantitative methods, or both, were included in the review, while research focusing on general practitioners and customers were excluded from the review. The search was also narrowed down to English language articles published in the last 12 years. After screening, the full-text articles were read, and information was abstracted by using a standardized data form in a table format.

In this process, three researchers independently studied and reviewed the abstracts of the papers and met three times (beginning, middle, and end) face-to-face to discuss the vague aspects and chose to redefine their strategy. Two other researchers read the full text of the articles for final inclusion. On strong disagreement of any study inclusion, the remaining researchers decided its final inclusion. 

### 2.4. Step 4: Data Charting

Based on Arksey and O’Malley’s framework, the studies were categorized based on the authors; publication year; type of study; study tool; study population; study location; aims and objectives of the study; and study findings and conclusion and limitations [31].

### 2.5. Step 5: Analyzing, Summarizing, and Reporting Results

A few stages were carried out in this step. After searching the studies using the databases, any unrelated literatures based on their titles and abstract were excluded. The full texts of the remaining studies were retrieved and read. The studies that did not fulfil the requirement for the review were excluded. The last 13 remaining studies were categorized based on their methodology, either using questionnaires or interviews, or using simulated patients/simulated clients.

The purpose of including simulated patient/simulated client studies stems from the fact that they are simply a direct reflection of pharmacists’ attitudes towards antibiotic dispensing and facilitates the validation of their reported perception and attitude in cross-sectional studies.

## 3. Results

**Figure 1:**
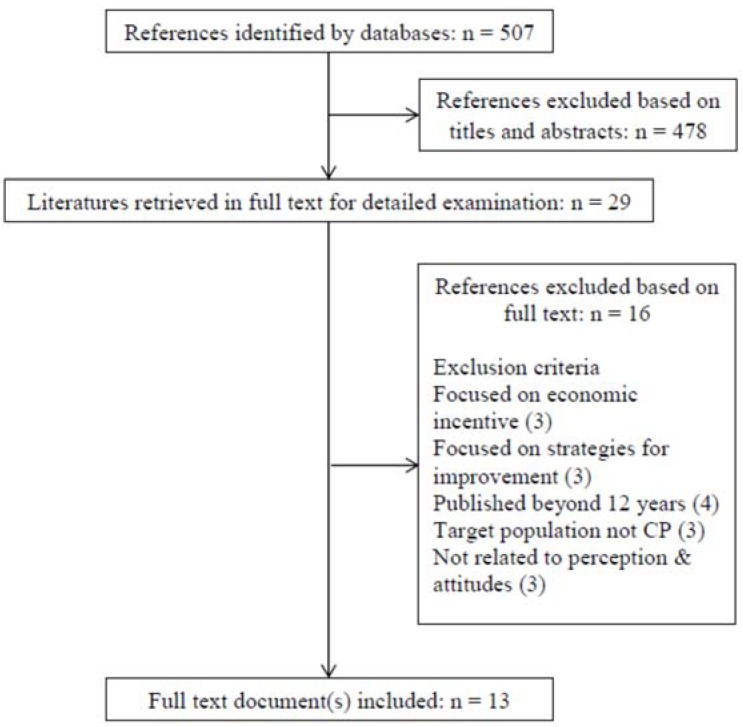
Quorum flow chart.

A total of 507 references were identified from the electronic searches of four databases after duplicates were removed. A total of 478 identified studies were excluded based on the titles and abstracts. From the 29 research articles retrieved for detailed examination 16 were excluded (Figure 1). A total of 13 full-text articles were finally decided to be included in the review.

### 3.1. Awareness and Perception towards Antibiotic Dispensing

In general, community pharmacists showed appropriate knowingness and perception towards antibiotic dispensing without prescription and all identified this as growing public health issue [16,17,18,25,28]. This is followed by diminished efficacy of antibiotics and treatment failure [17] leading to increased resistance [18]. According to Dillip et al., a large majority perceived their outlets as a place for the customers to obtain medical services and devices which motivate them to dispense antibiotics to the customers [17]. The research evidence from Spain acknowledged patient gratification over potential AMR, and preferred to dispense antibiotics despite their proper perception towards AMR [19]. However, most of the community pharmacists agreed that irrational antibiotics usage is one of the main causes of increased antibiotic resistance, and that all healthcare professionals need to think rationally and stop prescribing and dispensing antibiotic excessively.

### 3.2. Attitudes of Pharmacists towards Antibiotic Dispensing

The attitudes of the majority of the pharmacists did not parallel with their awareness and perception and, interestingly, dispensing without medical prescription (DAwMP) is an extensive phenomenon seen in many community settings [16,17,18,19,20,21,22,23,24,25,26,27,28]. There is a plethora of facilitators that seem to be involved in DAwMP and the most important ones are highlighted below:

#### 3.2.1. Patient/Customer Demand

The community pharmacists put the onus on customers/patients for nonprescription dispensing irrespective of which strata of society they are from [16,17]. Community pharmacists also underlined that those patients who previously benefitted from any antibiotic and considered it a “panacea for all”, or require it as “travel medicine”, or are not able to pay the consultation fees of the doctors were generally more concerned in getting antibiotics [16,17,25,28]. A few also cited apprehensions of losing their loyal clientele if not succumbing to the demands/requests of their customers [17,18]. Complacency of patients was also reported in a couple of studies as the motivational factor to dispense without prescription [19,25,28]. 

#### 3.2.2. Revenue/Profit/Sales/Business Reasons

Community pharmacists are generally eager to boost their business turnover, and seek more revenue and higher profits. Studies from India [17,24,26], Ethiopia [16], and Tanzania [17] reported that refusal to dispense antibiotics can affect their sales and decrease their profit margin, followed by the accumulation of stock of expensive medicines.

#### 3.2.3. Doctor/Health Facility Prescription

There always exists a bond of mutual trust and confidence among patients and doctors. From research evidence from Tanzania, community pharmacists cited their inability to correct health facility prescriptions for nonpneumonia cough and nonbloody diarrhea because patients had intrinsic trust and confidence for their doctors and always want to adhere to doctors’ prescriptions [17]. Likewise, in a study from Portugal, community pharmacists dispense antibiotics to their clients because they understand that doctors, themselves, will prescribe antibiotics in clinical situations like tooth infections and urinary tract infections [25]. 

### 3.3. Commonly Dispensed Antibiotics

This review also highlighted that specific antibiotics were commonly dispensed to each scenario accounting for sore throat, URTI, sinusitis, urinary tract infection, acute bronchitis, otitis media, acute gastroenteritis, and/or diarrhea. Penicillin only or combination with penicillinase inhibitor were the most common antimicrobial agents dispensed for URTI [20,21,23,26,27]; the fluoroquinolone group was commonly dispensed for UTI scenario [21,23]; the fluoroquinolone group and antiprotozoan agents are mostly dispensed for diarrhea, either alone or in combination [20,23,24,26]; while for otitis media penicillin, chloramphenicol, or macrolides were dispensed [20,23]. Interestingly, in all these regions legal implications existed, but most of the dispensing attitudes were attributed to customers’ demands, gratification, and satisfaction, the pharmacy owner’s pressure on pharmacists or dispensing personnel, and/or financial motives of community pharmacists. Almost all of the dispensing attitudes deemed inappropriate and injudicious owed to the nature of the disease categorized under ‘minor ailments’ or ‘self-limiting’.

### 3.4. Simulated Patients/Simulated Clients/Voluntary Collaborators

The strategy of executing simulated patients in assessing the attitudes of community pharmacists is appropriate to quantify customer care. This concept is well-established in developing countries where it is considered pertinent and economical and helps in identifying areas of improvement for providing quality services in patient care [33]. The current review included simulated patient research from Spain, Greece, India, Malaysia, Jordan, and the Kingdom of Saudi Arabia (KSA) [20,21,22,23,24,26,27]. In the KSA study, 95% of antibiotics were dispensed on the pharmacist’s own discretion and not per the demand of patients [23]. On the contrary, studies from Spain and Jordan reflected clearly that more than 50% of patients requested antibiotics in minor and self-limiting conditions [20,21]. The study from Greece approached pharmacies through voluntary collaborators who directly request antibiotics and did not imposter themselves as simulated patients having validated clinical scenarios [22]. Even then, 85% of the pharmacists did not ask for any justification and dispensed without prescription. To be precise, community pharmacists were flexibly and willingly found dispensing antibiotics to simulated patients or voluntary community pharmacy collaborators. For details please refer to Table 1.

## 4. Discussion

Based on the review undertaken, most of the community pharmacists presented appropriate awareness towards AMR [16,17,18,19,20,21,22,23], but the majority reflected unacceptable attitudes towards dispensing and preferred to perform DAwMP in minor ailments and self-limiting conditions [16,17,18,19,20,21,22,23,24,25,26,27,28]. The majority of the papers included in the review identified several enablers that promote DAwMP, such as indifference on the part of pharmacists, fulfilment of the demands of patients/clients/customers, external responsibility, and insufficient knowledge of the consumer and/or patient [16,17,18,19,20,21,22,23,24,25,26,27]. The knowledge and awareness of pharmacists on disease management was predictably outstanding [16,18] since they are considered experts with respect to medicines. Additionally, the pharmacists need to be continually vigilant not only towards the development and issues in healthcare for providing the best treatment to their customers, but also towards the preventive measures that can intervene with the patient’s recovery, such as AMR. Inappropriate knowledge, understanding, and awareness on AMR can increase unnecessary antibiotic dispensing [19], and further worsen AMR. A steady knowledge and consistent understanding on the antibiotics and AMR among the community pharmacists pave the way towards reduced AMR progression through improved adherence towards antibiotic usage and reducing self-medication [18,34], and also channelize the injudicious demands of antibiotics from the community pharmacy [16].

URTI, the most commonly reported case at primary care institutions, are mostly of viral origin. In the current review the URTI scenarios used viral cough as the presented problem and the community pharmacists always dispensed penicillin or penicillin plus penicillinase inhibitor as the treatment [20,21,23,26,27]. The staggering fact is that in these cases community pharmacists dispensed antibiotics. On the other hand, UTIs stem from bacterial infection more frequently in women. Community pharmacists dispensed fluoroquinolones to their simulated patients/clients [20,21,23] without proper history-taking. The importance of history-taking in community pharmacies cannot be denied as this can help in evading common pitfalls, such as in the case of category C medicines, like fluoroquinolones, which have teratogenic effects and must be dispensed with caution in women [35]. We observed in our review that most of the pharmacists did not assess the simulated patients (SPs) precisely and dispensed antibiotics as per their demand.

Otitis media is one such condition in which antibiotic intervention is generally not recommended [36]. Nevertheless, the common action observed among community pharmacists was to dispense amoxicillin/clavulanic acid and chloramphenicol [20,23]. For diarrhea, the nature of the problem needs to be diagnosed based on patient’s history as its etiology is frequently attributed to food poisoning and, therefore, antimotility agents are therapeutically effective. The frequent usage of antibiotics in paediatric diarrhea can be a potential contributory factor to AMR [24]. 

The current review reported a liberal attitude of community pharmacists towards antibiotic dispensing in all the simulated clinical scenarios. Especially in case of URTI, it was observed that antibiotics were given mostly without emphasizing the demand from the SPs [20,21,22,23,26].

A recently published commentary pinned high hopes on community pharmacists and positioned them as antibiotic stewards in upper respiratory tract infections [37]. Likewise, both the studies from Portugal [25,28] highlighted that attitudes of community pharmacists towards DAwMP are driven by patient-related factors and educational interventions directed towards improved patient–pharmacist interactive sessions are needed for exercising appropriate and rational use of antibiotics.

Unlike the current scoping review, the systematic review by McCullough et al. [38] focused mainly on clinicians and reported that clinicians considered antimicrobial resistance a thought-provoking matter, but the responsibility mainly lies on patients or other healthcare providers.

## 5. Conclusions

The majority of the community pharmacists reflected necessary knowledge and appropriate perceptions toward antibiotic dispensing and resistance. The majority acknowledged that antimicrobial resistance is the culmination of injudicious and excessive antibiotic dispensing and irrational usage. However, antibiotics are still dispensed freely in a community setting and, thus galvanizing widespread antibiotic resistance. The authorities should reflect intensified mindfulness towards this problem by strengthening the implementation of current regulations, followed by brainstorming new alternatives to curb this menace. 

Finally, community pharmacists can work as collaborators between the prescribers and patients, educating patients on ‘where’, ‘when’, and ‘how’ to use antibiotics effectively, as well as to remind prescribers to follow guidelines, and furnishing them with up-to-date information about different therapeutic categories. Mass media campaigns voiced over by community pharmacists on the disposal of unused antibiotics can be one such future strategy which needs to be implemented soon in lower- and middle-income countries.

## Figures and Tables

**Table 1:** Summary of studies of perceptions and attitudes of antibiotic dispensing.

Author, Year, [Ref.]	Study Type Study Tool	Study Participants Study Site Sampling	Aims/Objectives	Study Findings	Conclusion Limitation
Dillip, 2015, [17]	Qualitative; In-depth interviews	Accredited drug dispensing outlet (ADDO) owners and dispensers; Tanga and Ruvuma; Tanzania purposive interviews (7)	Exploring attitudes towards antibiotic dispensing; Accreditation influence on dispensing	Good knowledge but low implementation; Customer demand Profit margin; Habit to follow doctors’ prescriptions	Positive influence of ADDO Program but absence of translation into practice; Nongeneralizable sample Social desirability bias
Zapata, 2014, [19]	Cross-sectional Self-administered questionnaire	286 Community pharmacists (CPs) Spain Exhaustive sampling	Interpretation of knowledge and attitudes for DAwMP	Positive relationship with DAwMP; Indifferent attitude, complacency; Insufficient knowledge	Strong relationship with DAwMP; Nonresponse bias; Inappropriate validity criterion
Gebretekle, 2016, [16]	Phenomenological qualitative; In-depth interviews Observation	Five CPs Ethiopia	Exploring reasons—OTC antibiotics sales	Frequent DAwMP; Weak enforcement regulation; Customer demand, profit margin	OTC antibiotic sales-common practice; Strict regulatory enforcement; Educational campaigns; Non-generalizable sample
Hadi, 2015, [18]	Cross-sectional; Self-administered questionnaire	189 CPs Makkah, Saudi Arabia	Exploring knowledge, attitude, practices towards DAwMP	Insufficient knowledge of legality; Knowledgeable of consequences of DAwMP Patient unwillingness to consult doctors; Unaffordability of doctors’ consultations	Ignorant of DAwMP as illegal Educational interventions; Single study setting (Makkah); Non-generalizable; Social desirability bias
Almaytah, 2015, [20]	Prospective design; Five clinical scenarios (SPs)	202 pharmacies Jordan	Assessing knowledge of viral symptoms; DAwMP	Unnecessary dispensing for sore throat and UTI; Insufficient knowledge of duration of treatment; Antibiotic dispensing refusal	Absence of abidance of national regulation
Llor, 2009, [21]	Prospective design; Three clinical scenarios (SPs)	197 pharmacies Spain	Exploring the attitude for DAwMP; Quantifying the extent of DAwMP	Unnecessary dispensing for sore throat and acute bronchitis; Recommended consultation	Though illegal DAwMP observed; Not confirmed whether community pharmacist dispensing
Plachouras, 2010, [22]	Prospective design; 21 voluntary collaborators	174 pharmacies Greece	Quantifying the extent of DAwMP	Huge and easy dispensing Amoxicillin/clavulanate and ciprofloxacin largely requested	Despite implementation of restriction ciprofloxacin dispensed; Educational strategies for pharmacists; Need of strong regulation enforcement
Abdulhak, 2011, [23]	Cross-sectional; SPs	327 pharmacies Saudi Arabia Quasi-random	Exploring DAwMP; Exploring associated risks	DAwMP observed without patients’ requests for sore throat and diarrhea; Improper history-taking	DAwMP routinely observed; Requirement of stringent enforcement and compliance to regulations
Alabid, 2014, [27]	Cross-sectional; SPs	50 pharmacies Malaysia Convenient	Investigating antibiotic dispensing for (upper respiratory tract infections) URTIs	Noncompliance rational use of drug concept (RUD) by WHO	Irrational antibiotic dispensing; Polypharmacy; Nonrepresentative sample
Diwan, 2015, [24]	Cross-sectional; Simulated clients (SCs)	164 pharmacies India	Investigating treatment of childhood diarrhea	DAwMP with antimotility agents and probiotics; Actual treatment (oral rehydration salt)	Highly inappropriate treatment; Actual treatment under-dispensed; Not confirmed whether community pharmacist dispensing
Shet, 2015, [26]	Cross-sectional; Two clinical scenarios (SPs)	261 pharmacies India	Exploring frequency of DAwMP	Frequent DAwMP; Inappropriate management for URTI and self-limiting illnesses	Highly undesirable DAwMP; Not confirmed whether community pharmacist dispensing
Roque, 2013, [25]	Exploratory qualitative; Semi-structured interviews (six focus groups)	32 CPs Portugal	Exploring the knowledge, perception and attitude	Knowledgeable about rational dispensing and antibiotic resistance; Factors: Physicians and patients and veterinary consumption for antibiotic resistance	Fair improvement chances; Behavioral intention for expected change; Non-generalizable sample; Chances of bias in FGD
Roque, 2015, [28]	Cross-sectional; Self-administered questionnaire	770 CPs Portugal	Evaluating DAwMP; Attitude towards DAwMP	Frequent DAwMP-dental ailments and UTIs; Factors: Patient satisfaction, precaution, and fear for DAwMP	Attitudes promotes DAwMP; Educational interventions; Nonresponse bias; Social desirability
